# Case Report: Two cases of chronic active Epstein-Barr virus disease presenting as refractory sinusitis

**DOI:** 10.3389/fimmu.2025.1678519

**Published:** 2025-10-02

**Authors:** Puyu Wang, Dong Dong, Shuman Huang, Linyuan Wang, Hui Zhang, Yuanyuan Li, Yulin Zhao

**Affiliations:** ^1^ Department of Otolaryngology Head and Neck Surgery, The First Affiliated Hospital of Zhengzhou University, Zhengzhou, China; ^2^ Department of Oncology, The First Affiliated Hospital of Zhengzhou University, Zhengzhou, China

**Keywords:** chronic active Epstein-Barr virus disease, refractory sinusitis, lymphoproliferative disease, chimeric antigen receptor T-cell immunotherapy, case report

## Abstract

**Background:**

Chronic active Epstein-Barr virus disease (CAEBV) is a systemic, progressive lymphoproliferative disorder caused by the Epstein-Barr virus (EBV) infecting T cells and/or natural killer (NK) cells. It exhibits both inflammatory and clonal proliferative characteristics. Given the rarity of this disease, there is a relative scarcity of diagnostic and therapeutic expertise. Additionally, the clinical manifestations are varied and affect multiple systems, which can lead to cases being either missed or misdiagnosed. Consequently, the prognosis is often poor.

**Case presentation:**

This article reports two cases of systemic CAEBV presenting as refractory sinusitis. Case one is an 18-year-old male, and case two is a 22-year-old female. The main symptoms of both patients were intermittent fever, nasal congestion and bloody nasal discharge for more than six months. Pathological examination indicated EBV-associated lymphoproliferative disease. The diagnosis of systemic CAEBV was established on the basis of clinical presentation and pathological findings and EBV DNA test. Both patients showed poor response to immunotherapy or chemotherapy and progressed to hemophagocytic lymphohistiocytosis (HLH). Case one was eventually cured by Chimeric Antigen Receptor T-Cell Immunotherapy (CAR-T). In case two, the condition deteriorated rapidly and she died of multiple organ failure and septic shock after hematopoietic stem cell transplantation.

**Conclusion:**

This rare case report describes two cases of systemic CAEBV presenting as refractory sinusitis. The report also shows the potential of CAR-T as a treatment for CAEBV. We comprehensively reviewed the diagnostic and therapeutic trajectories of both patients from disease onset, hoping to provide reference for clinical practice.

## Introduction

With over 90% of the population having experienced EBV infection and carrying it lifelong (1), CAEBV is classified within the family of “EBV-positive T- and NK-cell lymphoid proliferations.” This category is relatively rare and primarily affects T-cell and NK-cell diseases, predominantly observed in Asian and native American ethnic groups (2). CAEBV presents with a variety of clinical manifestations and lacks specificity. It can be classified as cutaneous (localized/inactive) or systemic, depending on the extent of its involvement. The clinical course of CAEBV is diverse; some patients exhibit slow progression and remain stable over several years, while others present with aggressive disease, which may lead to hemophagocytic lymphohistiocytosis, multi-organ failure, or leukemia and lymphoma (3, 4). CAEBV was originally considered a childhood disease; however, recent studies have identified a growing number of adult cases and a worse prognosis compared to that in children (5).

In this research, we report two cases with early symptoms similar to sinusitis, with a long duration from onset to diagnosis, confirmed only after multiple pathological examinations at different hospitals. Cases in the field of otolaryngology surgery are relatively rare, and clinicians have limited awareness of diagnosis and treatment, making diagnosis challenging. We reviewed their clinical data with the aim of accumulating experience for the diagnosis and treatment of this disease. This case report was written in accordance with the CARE (Case Reports) guidelines (https://www.care-statement.org/checklist).

## Case presentation

### Case 1

An 18-year-old Chinese male presented to our hospital in December 2022 with intermittent fever, nasal congestion and bloody nasal discharge, symptoms that had persisted for four years. The patient was diagnosed with sinusitis and underwent functional endoscopic sinus surgery one year ago; however, the symptoms continued to recur. Three months ago, he was diagnosed with secretory otitis media due to left ear stuffiness, hearing loss, and dizziness, and underwent surgery. The patient had no underlying diseases, and there was no family history of similar or genetic diseases.

**Figure 1:**
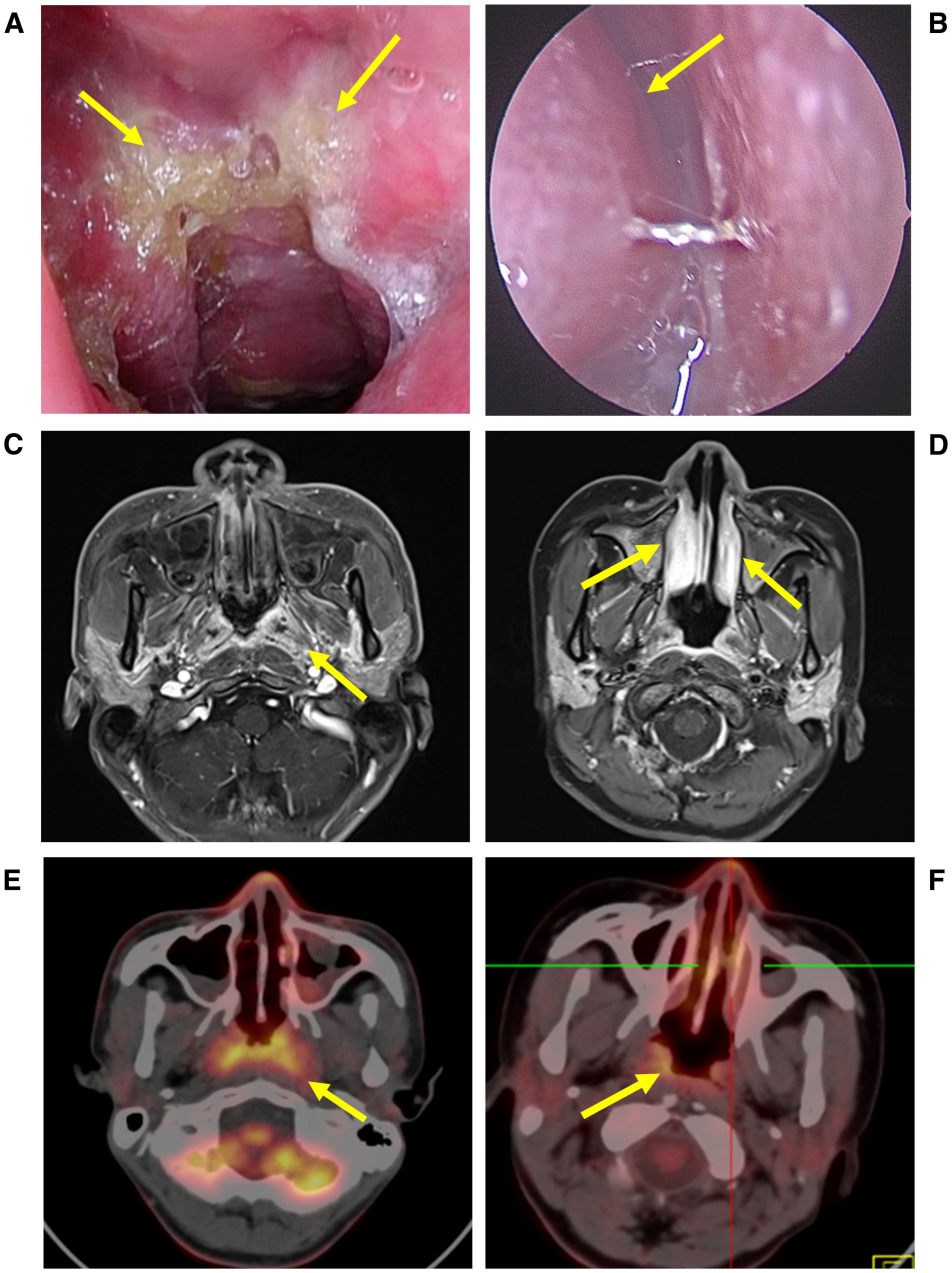
Nasal endoscopy and imaging findings of the two patients. **(A)** Nasal endoscopy of the patient revealed lymphoid hyperplasia in the nasopharynx, the pharyngeal recess and the torus tubails were indistinct. **(B)** Inferior turbinate swelling with retention of secretions. **(C)** Enhanced MRI: 30% thickening of soft tissue on the posterior wall of the nasopharynx, mild to moderate enhancement after intravenous injection of contrast agent. Shallow bilateral pharyngeal recess. Inflammatory effusion in bilateral maxillary sinuses. **(D)** 40% thickening of the right inferior nasal turbinates, inflammatory effusion of bilateral maxillary sinuses. No significant thickening of soft tissues in the nasopharynx, and no significant enhancement in the nasal cavity enhanced scan. **(E)** The mucosal thickening of the lateral and posterior walls of the nasal cavity exhibited a concentrated distribution of radioactivity (SUVmax approximately 10.8). **(F)** Slightly thickened radioactivity distribution in the nasopharynx (SUVmax about 8.3). A small soft tissue shadow was seen in both nasal cavities with relatively concentrated radioactivity distribution (SUVmax about 6.3).

**Figure 2:**
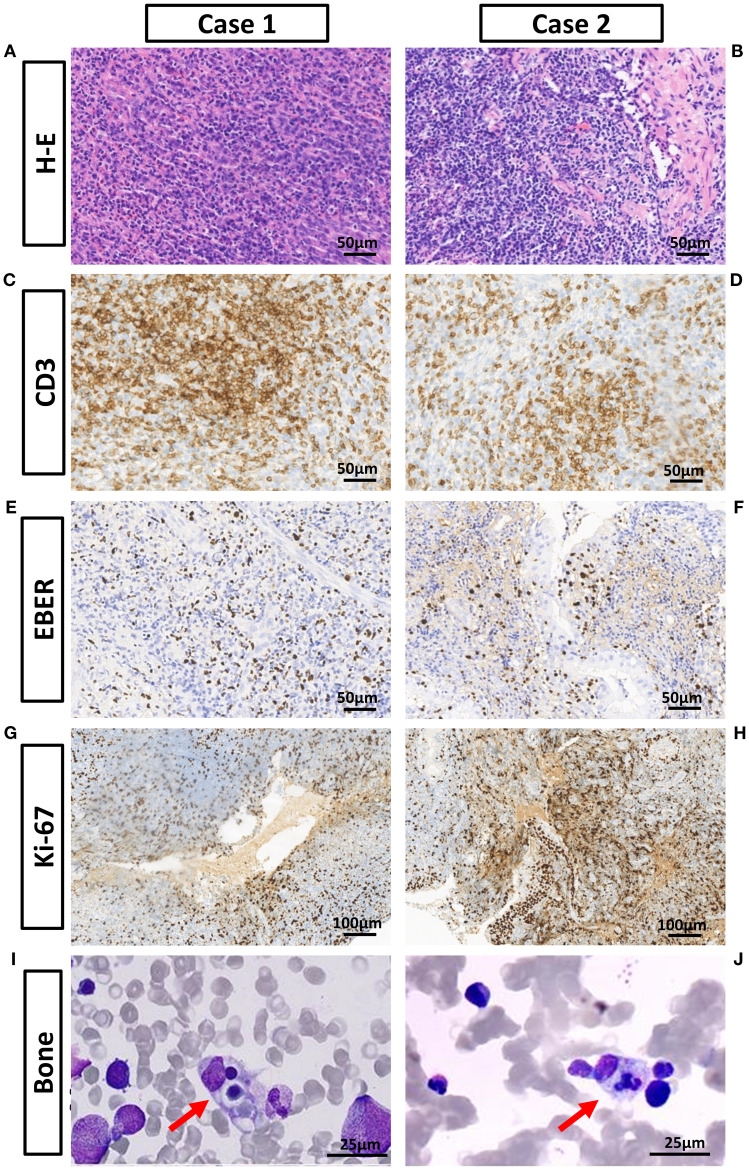
Pathological examination results of the nasal cavity, nasopharynx, and bone marrow aspiration. **(A, B)** Hematoxylin-Eosin staining: Abundant lymphocytic infiltration with mild atypia is observed in both specimens. **(C, D)** CD3 staining is positive in both specimens. **(E, F)** EBER *in situ* hybridization is positive in both specimens. **(G, H)** The Ki-67 index is approximately 40% and 30%. **(I, J)** Hemophagocytic phenomenon was observed in both bone marrow smears under high-power microscopy.

Physical examination showed no enlargement of superficial lymph nodes, and no jaundice, rash or petechiae on the skin and mucous membranes. Nasal endoscopy revealed lymphoid hyperplasia in the nasopharynx, the pharyngeal recess and the torus tubails were indistinct (**Figure 1A**). Enhanced magnetic resonance imaging (MRI) indicated 30% thickening of the soft tissue of the posterior wall of the nasopharyngeal roof and shallow pharyngeal recess bilaterally (**Figure 1C**). Given the patient’s prolonged fever, a comprehensive blood examination was conducted. The patient tested positive for whole blood EBV DNA and exhibited mild hepatic dysfunction and anemia. A nasal endoscopic biopsy of nasopharyngeal tissue indicated EBV-associated T-lymphocyte proliferative disease, grade I (reactive) (**Figures 2 A, C ,E, G**). Immunohistochemistry was positive for CD3, CD21, Bcl-2, T-cell intracellular antigen-1 (TIA-1); partially positive for CD5, CD20, CD79α, CD10, Bcl-6, CD56, and granzyme B; negative for CyclinD1, CD30, and perforin. The Ki-67 index was approximately 40%; *in situ* hybridization was positive for Epstein-Barr virus-encoded small RNA (EBER)(**Supplementary Figure 2**). Fluorodeoxyglucose-Positron Emission Tomography/Computed Tomography (FDG PET/CT) demonstrated mucosal thickening of the nasal pharyngeal dorsolateral wall and bilateral walls with concentrated radioactivity (**Figure 1E**). Based on the patient’s medical history and various examination results, systemic CAEBV was diagnosed.

From January 2023, the patient received PD-1 monoclonal antibody Tislelizumab 200mg twice, 28 days apart. During treatment, he still experienced intermittent fever, with body temperature fluctuating 36.4-39.0°C, 1–2 daily fever peaks. Therefore, in February 2023, we performed a second biopsy on him, taking samples from the nasopharynx and the left inferior turbinate. It still showed EBV-associated T-lymphocyte proliferative disease, but progressed to grade II (borderline) (**Supplementary Figure 3**). Immunohistochemistry showed positivity for CD2, CD3, CD4, CD5, Bcl-2, TIA-1; partial positivity for CD7, CD20, CD38, CD79a, CD138, Bcl-6, Kappa, Lambda, Granzyme B, and multiple myeloma oncogene 1 (MUM-1); negativity for AE1/AE3, CD8, CD10, CD21, CD30, CD56, CyclinD1, with Ki-67 index>20%. EBER *in situ* hybridization was positive, and TCR gene rearrangement was monoclonal. We recommend that patients be switched to the DDGP regimen, which consists of gemcitabine (1.2g, d1,d5), cisplatin (30 mg, d1-d4), pegaspargase (3750 IU, d1), and dexamethasone (25 mg, d1-d4), once every three weeks. After 3 cycles of chemotherapy, the patient still had persistent high fever, peaking at 40.4°C.

During this period, laboratory examination indicated pancytopenia, and levels of triglycerides, ferritin, and soluble CD25 were elevated (**Table 1**). Bone marrow aspiration revealed the presence of hemophagocytic macrophages (**Figure 2I**). Abdominal ultrasound showed diffuse hepatomegaly. Blood metagenomic Next-generation sequencing (mNGS) revealed that EBV was detected at sequence position 247, with a relative abundance of 99.6% and a coverage of 4.36%. Based on the HLH-2004 criteria, the patient was diagnosed with HLH. Treatment for HLH was initiated with etoposide and dexamethasone. The patient declined allogeneic hematopoietic stem cell transplantation (allo-HSCT) for personal reasons. After consulting with family members, we recommend CAR-T cell therapy. The patient underwent a single nucleocyte collection in early June 2023, followed by low-dose CHOP bridging chemotherapy. On the sixth day following CAR-T cell infusion, the patient’s temperature normalized, and EBV DNA levels dropped below normal. As the patient’s indices gradually normalized, we discharged the patient and advised regular examinations of whole-blood EBV DNA, nasal endoscopy, and blood routine.

**Table 1:** Laboratory test results of the patients.

Test item	Unit	Case 1 pre-CAR-T	Case 2 pre-transplant	Case 2 post-transplant	Normal range
RBC	*10^12/L	3.37	2.05	1.62	Male: 3.5-5.5 Female: 3.0-5.0
WBC	*10^9/L	2.34	0.50	2.10	4-10
Platelets	*10^9/L	241	16	3	100-300
Hemoglobin	g/L	89	76	88	>110
Triglycerides	mmol/L	1.88	2.43	2.33	<1.7
Fibrinogen	g/L	1.30	0. 89	1.02	2-4
Ferritin	ng/mL	3558.50	1657. 00	1433.00	13-150
Soluble CD25	pg/ml	1508.36	742.09	unavailable	26-314
NK cell count	/μL	45	21.69	22.40	150-1100
Whole blood EBV DNA	IU/ml	98644	3644	Negative	Negative^*^

*The negative range should be referenced to the specific test device specification.

**Figure 3:**
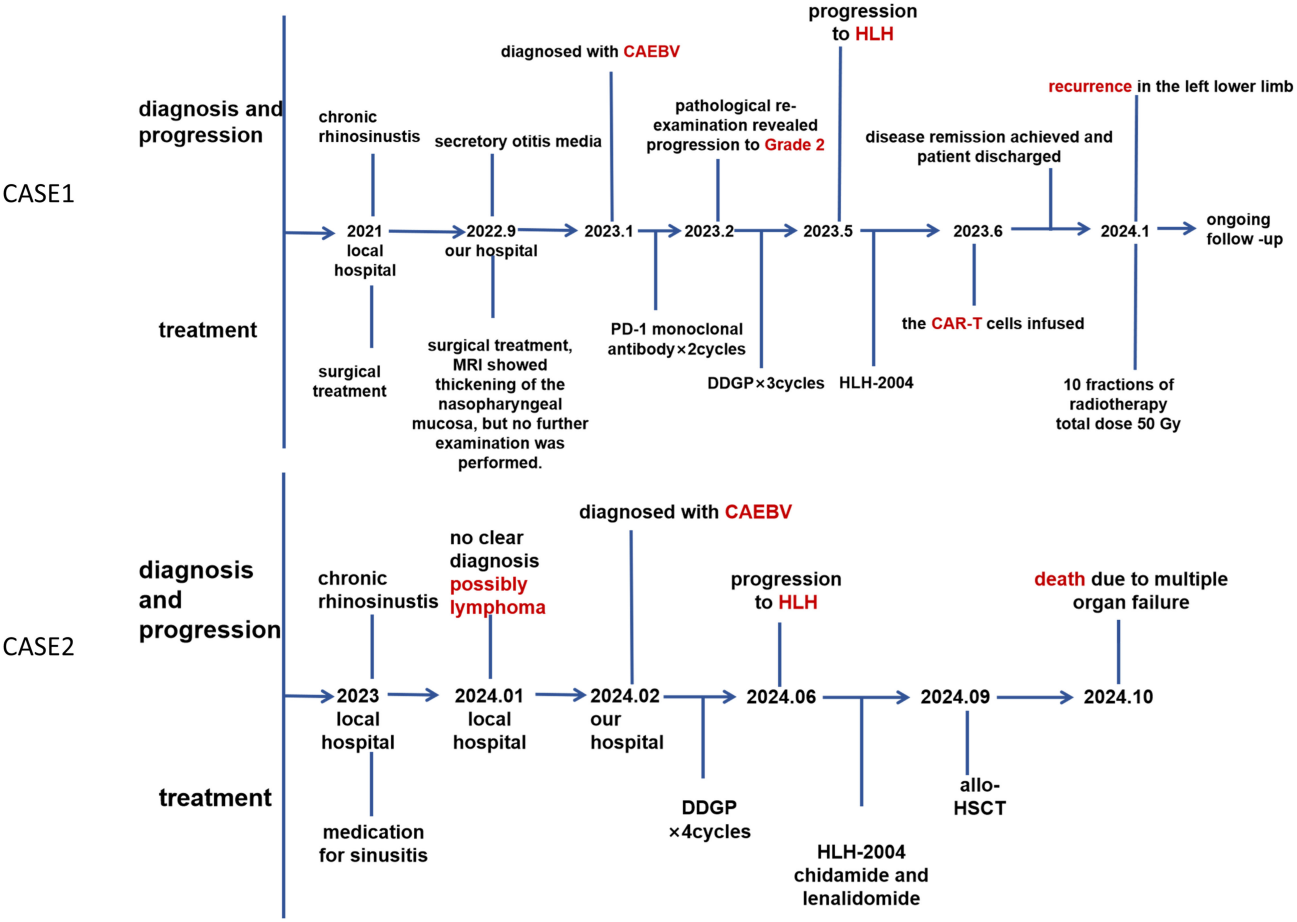
Timeline for the clinical events and treatment.

Three months after being after discharged, the patient developed pain in the left lower limb. Pathological examination results indicated that it was still an EBV-associated lymphoproliferative disease. Subsequently, the patient underwent a total of 10 radiation therapy sessions to the left pelvis and left lower extremity, amounting to 50 Gy. The pain in the left lower limb then subsided. Oral etoposide at a dosage of 0.1 g per day was administered as maintenance therapy. The patient has been monitored in the outpatient clinic for 17 months without any recurrence, and the whole blood EBV DNA remains undetectable. At present, he is in good mental state and has started college life. The timeline shows the treatment process and clinical findings since the onset of the disease (**Figure 3**).

### Case 2

A 22-year-old Chinese female presented to our hospital in February 2024 with intermittent fever and blood in the mucus for six months. The highest recorded body temperature was 39.3°C, accompanied by headache, bilateral nasal congestion, and hyposmia. Initially, she was diagnosed with sinusitis at a local hospital and treated with nasal spray hormones and oral medication, but the treatment proved ineffective. Subsequently, a biopsy of the left inferior turbinate was taken at the local hospital, which suggested interstitial lymphoid hyperplasia and suspected lymphoma. The patient had no underlying diseases, and there was no family history of similar or genetic diseases.

Physical examination revealed no enlargement of superficial lymph nodes, and no jaundice, rash, or petechiae on the skin or mucous membranes. Nasal endoscopy revealed swelling of the inferior turbinates (**Figure 1B**). Enhanced MRI showed no significant thickening of the soft tissue in the nasopharynx (**Figure 1D**). Multiple biopsies from the bilateral inferior nasal conchae, nasal floor, and nasal septum revealed an EBV-associated T/NK-lymphocyte proliferative disease, at least grade 2 (**Figure 2B, D, F, H**). Immunohistochemistry results were as follows: AE1/AE3, CD3, CD4 positive; CD5, CD8, CD19, CD20, CD30, CD56, P53, TIA-1, Granzyme B partially positive; CD21 negative; Ki-67 index >30% (**Supplementary Figure 4**). EBER *in situ* hybridization was positive, and TCR gene rearrangement was monoclonal. FDG PET/CT indicated slight thickening and concentrated radiation distribution in the nasopharynx (thickened by 30%) and bilateral nasal cavity (**Figure 1F**). The patient was ultimately diagnosed with systemic CAEBV. Additionally, a timeline is displayed to show the progression of these treatment processes and clinical findings (**Figure 3**). Subsequently, the patient underwent the DDGP chemotherapy regimen, which consisted of gemcitabine (1g, d1 and d5), cisplatin (25mg, d1 to d4), pegaspargase (3750IU, d1), dexamethasone (20mg, d1 to d4), once every three weeks. Throughout this period, intermittent high fevers occurred, with body temperatures ranging from 36.6 to 39.2°C. After four cycles of chemotherapy in June 2024, the efficacy evaluation revealed disease progression (PD). Laboratory tests indicated pancytopenia with elevated ferritin, soluble CD25, and triglycerides (**Table 1**). Hemophagocytic cells were observed in the bone marrow aspiration smear (**Figure 2J**). Blood mNGS revealed that EBV was detected at sequence position 1377, with a relative abundance of 92.1% and a coverage of 31.3%. Based on the HLH-2004 criteria, the patient was diagnosed with hemophagocytic lymphohistiocytosis. Treatment for HLH was initiated with etoposide (150mg, q4d) and dexamethasone (15mg, qd). In July 2024, the chemotherapy regimen was altered to sidabenamide (20mg, q3d) and lenalidomide (25mg, qd). However, the condition continued to deteriorate, with the blood cell count dropped to a critical level. After consulting the hematology department, we recommended allogeneic hematopoietic stem cell transplantation (allo-HSCT). However, due to financial reasons, the patient didn’t undergo the transplant until three months after the disease progressed to HLH. The treatment regimen prior to transplantation included whole body irradiation, cyclophosphamide, and Thiotepa. Anti-rejection therapy comprised cyclosporine, mycophenolate mofetil, methotrexate, anti-thymocyte immunoglobulin, and sirolimus.

The patient developed persistent high fever (peak temperature 39.4°C) post-transplantation, accompanied by severe anemia and progressive deterioration of hepatic, renal, and coagulation functions. Metallo-β-lactamase-producing carbapenem-resistant Escherichia coli was detected in the blood culture. Clinical manifestations included jaundice, skin ecchymosis, and pleural, peritoneal, and perisplenic effusions. The diagnosis of acute graft-versus host disease (GVHD) is insufficient due to the lack of biopsy specimens. The patient unfortunately passed away in October 2024 due to multiple organ failure and septic shock.

## Discussion

EBV-infected cells become active, proliferate, and infiltrate various organs, causing systemic inflammation associated with a cytokine storm (6). In addition to infectious mononucleosis-like symptoms, CAEBV can be manifested in any area of cellular infiltration. Current literature indicates that nasal and pharyngeal tissues are more commonly affected than other sites in cases of CAEBV and tend to present as refractory sinusitis (7–10). This phenomenon is closely related to the biological characteristics of EBV, the immune microenvironment, inflammatory cascade and the anatomical features of the rhinosinus. EBV primarily spreads through saliva, initially infecting epithelial cells in the oropharynx and B lymphocytes upon entry into the human body (11, 12). The upper respiratory tract is rich in lymphoid tissues, including Nasal-associated lymphoid tissue (NALT) and Waldeyer’s ring, which are important components of the body’s first line of defense against foreign pathogens (13). However, they also provide a suitable environment for EBV colonization. Furthermore, the pseudohierarchical ciliated columnar epithelium in the upper respiratory tract contains numerous crypts and folds, which increase the surface contact area and facilitate viral colonization. During upper airway inflammation, local inflammatory factors such as Interleukin (IL) -6, Tumor Necrosis Factor (TNF) -α, and Interferon (IFN) -γ can accelerate the transition of EBV from latency to the lysis phase. Simultaneously, IFN-γ upregulates chemokine ligands C-X-C motif chemokine ligand (CXCL)9, CXCL10, and CXCL11, leading to the mucosal homing of C-X-C motif chemokine receptor (CXCR) 3-expressing T/NK cells (14–16). The osteomeatal complex (OMC), a common drainage channel for the anterior group of sinuses, is narrow. Mild mucosal edema or anatomical abnormalities can cause obstruction of ventilation and drainage, leading to chronic inflammation. This may be why cases involving nasal or pharyngeal sinuses often present as refractory sinusitis (17).

The diagnosis of CAEBV necessitates fulfilling the following criteria: (1) Persistent or recurrent infectious mononucleosis-like symptoms (such as fever) lasting over three months; (2) Elevated EBV genome copies in peripheral blood or affected tissues; (3) EBV infection in peripheral blood or affected tissues involving T and/or NK cells; (4) Exclusion of other chronic diseases that may cause similar symptoms (such as autoimmune diseases, immunodeficiency, malignant tumors, etc.) (7). The clinical presentation, imaging and pathological results, and EBV DNA load of both patients supported the diagnosis of CAEBV. The most common differential diagnosis for CAEBV lesions in the nasal and pharyngeal regions is various lymphomas, particularly extranodal NK/T-cell lymphoma(ENKTL). ENKTL is characterized by its highly invasive and destructive nature. It predominantly affects the midface, especially the nasal cavity, followed by the nasopharynx and paranasal sinuses. Obvious mucosal ulcers and granulation-like neoplasms can be seen at the lesion site. In severe cases, there may be bone destruction, and even perforation of the nasal septum and hard palate (18–22). This is the most intuitive difference between it and the local lesions of CAEBV. Detailed differential diagnosis is in the (**Supplementary Table 1**). In contrast to lymphoma, CAEBV is associated with relatively low FDG uptake. For patients diagnosed with CAEBV, elevated FDG uptake values suggest the occurrence of lymphoma (23, 24). Vasculitis, IgG4-related disease, granulomatous disease, cystic fibrosis and immunodeficiency can also be the potential causes of refractory sinusitis (8, 25, 26).

Due to the diversity of CAEBV symptoms, patients may seek treatment in different departments, which adds difficulty to diagnosis. Experienced otolaryngologists performing multi-site sampling during surgery, along with accurate diagnosis made by authoritative pathologists, is crucial for diagnosis. It also indicates that the comprehensive level of hospital and Multidisciplinary Team Collaboration (MDT) is vital for the diagnosis of rare diseases.

Patients with persistent or recurrent infectious mononucleosis-like symptoms should be highly suspected of EBV-related diseases, and EBV DNA testing should be performed in time. It should be noted that plasma or serum EBV DNA is often negative during inactive periods; in such cases, testing whole blood or peripheral blood mononuclear cells (PBMCs) is more meaningful (7, 27). A tissue biopsy should be taken from the lesion site if conditions permit. Positive EBER *in situ* hybridization is the core evidence, directly indicating that lymphoid proliferation is associated with EBV infection at the histological level. EBER and EBV-DNA have complementary diagnostic value, and the total survival and progression-free survival of patients with both EBV-DNA and EBER positive were significantly lower than those with single positive or double negative (28, 29). In pediatric cases, EBV DNA load is predictive of prognosis, but this is not the case in adults. A retrospective study of 54 adult patients and 75 pediatric patients found that regardless of the baseline viral load, the prognosis of adult-onset CAEBV is generally poor. Moreover, neither the type of infected cells (T cells vs. natural killer cells) nor the EBV DNA copy number in the blood can predict the overall survival rate (with P-values of 0.59 and 0.82, respectively). The delayed diagnosis and rapid progression of adult cases may overwhelm the predictive effect of viral load on prognosis. This means that once a diagnosis of CAEBV is confirmed, the prognosis is very serious regardless of the viral load (30). The two patients in this report had significantly different blood EBV DNA loads, but both experienced severe disease deterioration, and even the patient with a low load had a faster progression than the one with a high load.

Allogeneic hematopoietic stem cell transplantation is currently the only effective method for treating CAEBV and also the most effective method for HLH (3, 7). Japanese scholars have proposed a three-step strategy for transplantation. The first step (Cooling) is to control systemic inflammatory responses and hypercytokinemia through immunochemotherapy. The second step (Cytoreduction) is to use multi-drug chemotherapy to eliminate EBV-infected T/NK cells and reduce the viral load before transplantation. The third step (Reconstruction) is to perform transplantation after reduced-intensity conditioning to reconstruct the immune system. The 3-year overall survival (3y-OS) rate of CAEBV treated with allo-HSCT is 71.4 ± 12.1% in adult. Planned HSCT has been successfully performed in cases where the disease is stable or has low activity, with an 3y-OS rate of 90.9 ± 8.7%. In contrast, 3y-OS in patients with uncontrolled active disease is only 16.7 ± 10.8% (31). Patients with combined HLH are advised to control HLH first and then transplant after efficacy evaluation to achieve partial response (PR) (3). In our study, case two missed the optimal time for transplantation due to the high cost. When the opportunity for transplantation arises three months after the diagnosis of HLH, it is no longer possible to effectively control the condition. The high cost of allo-HSCT precludes many patients from undergoing the complete three-step strategy, which may be one of the factors contributing to the poor prognosis. Post-transplant monitoring is crucial, and vigilance should be maintained against graft-versus-host disease, EBV reactivation, or infections by other pathogens (32).

Other therapeutic approaches include antiviral drugs, cytotoxic drugs, JAK inhibitors, as well as cellular and immunotherapies (3, 33). Due to the economic constraints of most patients, chemotherapy remains the most commonly used treatment method at present. It can partially reduce the level of EBV DNA, improve symptoms and organ function. However, chemotherapy has limited ability to eliminate infected cells and often only provides temporary efficacy. Moreover, intensive chemotherapy may cause various complications and accelerate organ failure. After the condition of Case 2 worsened, we switched to a milder oral chemotherapy regimen, but it still failed to effectively curb the progression of the disease. For patients who cannot receive transplantation timely, further research is needed on how to adjust the treatment plan to delay disease progression. The latest expert consensus suggests that chemotherapy should only be used as a preconditioning regimen before allogeneic hematopoietic stem cell transplantation, and high-intensity chemotherapy regimens should be selected with caution (34). Based on the results of PET/CT (**Supplementary Figure 1**), although the nasal lesions of the two patients improved after receiving personalized chemotherapy, their general condition continued to deteriorate and progressed to HLH, which confirms the above view.

Case one showed poor response to both PD-1 monoclonal antibody and chemotherapy. Therefore, we advised the patient and their family to undergo allo-HSCT as early as possible, but no suitable donor was found. To promptly prevent the further deterioration of the patient’s condition, we turned to emerging cell therapies. CAR-T cells therapy involves engineering the patient’s T cells through genetic engineering techniques to express artificially synthesized receptors, thereby enabling them to recognize and attack specific antigens. Glycoprotein (gp) 350, encoded by the BLLF1 gene, is the most abundant glycoprotein in the EBV envelope. It initiates the first step of infection by binding to CD21 on the surface of B cells and plays an important role in immune evasion after infection (35, 36). The expression of gp350 in the patient’s peripheral blood lymphocytes was detected by flow cytometry, so gp350 was selected as the therapeutic target. Currently, *in vitro* and animal experiments have confirmed that gp350-targeted CAR-T cells can effectively kill EBV-hosting cells and control the disease (37). There has been a report on the use of gp350-targeted CAR-T therapy in one 14-year-old child with CAEBV, who achieved good efficacy without severe adverse reactions after treatment (38). CAR-T cell therapy offers a new treatment option for patients who have failed at least two lines of standard treatment or are unable to receive HSCT, but it is currently in clinical trials and its long-term efficacy has not been proven, so it has not been widely accepted as a first-line treatment.

If this disease cannot be intervened timely, it may progress to lymphoma or HLH. The latent protein LMP-1 of EBV can activate the NF-κB pathway and plays an important role in disease progression (39–41). In our study, two patients progressed to HLH during treatment, which is a life-threatening inflammatory syndrome. 30-50% of secondary HLH are associated with EBV, and malignant tumors (especially lymphoma), autoimmune diseases, and chemotherapy can also cause secondary HLH (42, 43). Additionally, the long-term complications of CAEBV are a concern, particularly cardiovascular disease and massive bleeding (10, 44). Feng et al. reported a case of death due to a giant coronary artery aneurysm related to EBV infection seven years after CAEBV remission (45). Consequently, for the first case in this study, we recommend long-term outpatient follow-up to prevent recurrence and bleeding of mucosal erosion in the pharynx. Fortunately, this patient has not exhibited any abnormalities thus far.

Treatment strategies for CAEBV have entered an era of precision medicine, guided by molecular subtyping. Nevertheless, the disease frequently leads to fatal systemic complications, treatment is expensive, and the overall prognosis remains poor, necessitating a continuous accumulation of diagnostic and therapeutic experience.

In this report, we have combined clinical data from two patients with relevant literature to summarize the experiences and lessons learned in treatment and diagnosis. Although this report provides a reference for medical literature, we must acknowledge several limitations. The small sample size and short follow-up period of this study restrict the generalizability of the results. The effectiveness and durability of CAR-T therapy in treating CAEBV require verification through prospective studies with larger sample sizes and longer follow-up periods to better define its superiority over traditional therapies. We will continue to follow up and report the long-term efficacy of this patient.

## Conclusion

Patients with refractory sinusitis should actively seek out possible causes. Meanwhile, CAEBV should be highly suspected in patients with persistent or recurrent infectious mononucleosis-like symptoms. Upon diagnosing CAEBV, it is imperative to recognize the progressivity of the disease early in the treatment process, promptly identify the risk of severe complications, and adapt treatment strategies accordingly. Long-term follow-up is essential to monitor for any long-term complications. This study also indicates the potential of CAR-T as an effective treatment for CAEBV, but the effectiveness of this strategy needs to be validated through more large sample clinical trials and prospective studies.

## Data Availability

The original contributions presented in the study are included in the article/**Supplementary Material**. Further inquiries can be directed to the corresponding author.
