# Correction: Case Report: Two cases of chronic active Epstein-Barr virus disease presenting as refractory sinusitis

**DOI:** 10.3389/fimmu.2025.1723521

**Published:** 2025-10-27

**Authors:** Puyu Wang, Dong Dong, Shuman Huang, Linyuan Wang, Hui Zhang, Yuanyuan Li, Yulin Zhao

**Affiliations:** ^1^ Department of Otolaryngology Head and Neck Surgery, The First Affiliated Hospital of Zhengzhou University, Zhengzhou, China; ^2^ Department of Oncology, The First Affiliated Hospital of Zhengzhou University, Zhengzhou, China

**Keywords:** chronic active Epstein-Barr virus disease, refractory sinusitis, lymphoproliferative disease, chimeric antigen receptor T-cell immunotherapy, case report

In the published article, Reference 20 was incorrectly listed as “Zhang J, Jiang Q, Na S, Pan S, Cao Z, Qiu J. Retraction notice to: Minimal Scar Dissection for Partial Parotidectomy via a Modified Cosmetic Incision and an Advanced Wound Closure Method. J Oral Maxillofac Surg. (2022) 80:967. doi: 10.1016/j.joms.2022.03.001”.

The correct reference should be: “Zhou Z, Shao L, Zhang W. Progress in clinical diagnosis and treatment of chronic active Epstein-Barr virus infection. J Clin Hematol. 2022;35(1):11-5. doi: 10.13201/j.issn.1004-2806.2022.01.003.”.

The original version has been updated.

