# Supplementary material for: Case Report: Two cases of chronic active Epstein-Barr virus disease presenting as refractory sinusitis
Source: Front Immunol. 2025 Oct 2;16:1678519. doi: 10.3389/fimmu.2025.1678519 (PMC12528027; doi:10.3389/fimmu.2025.1678519)
Supplement: Supplementary file 1 [file Table1.docx]

Figure 1: FDG PET/CT images of two patients.
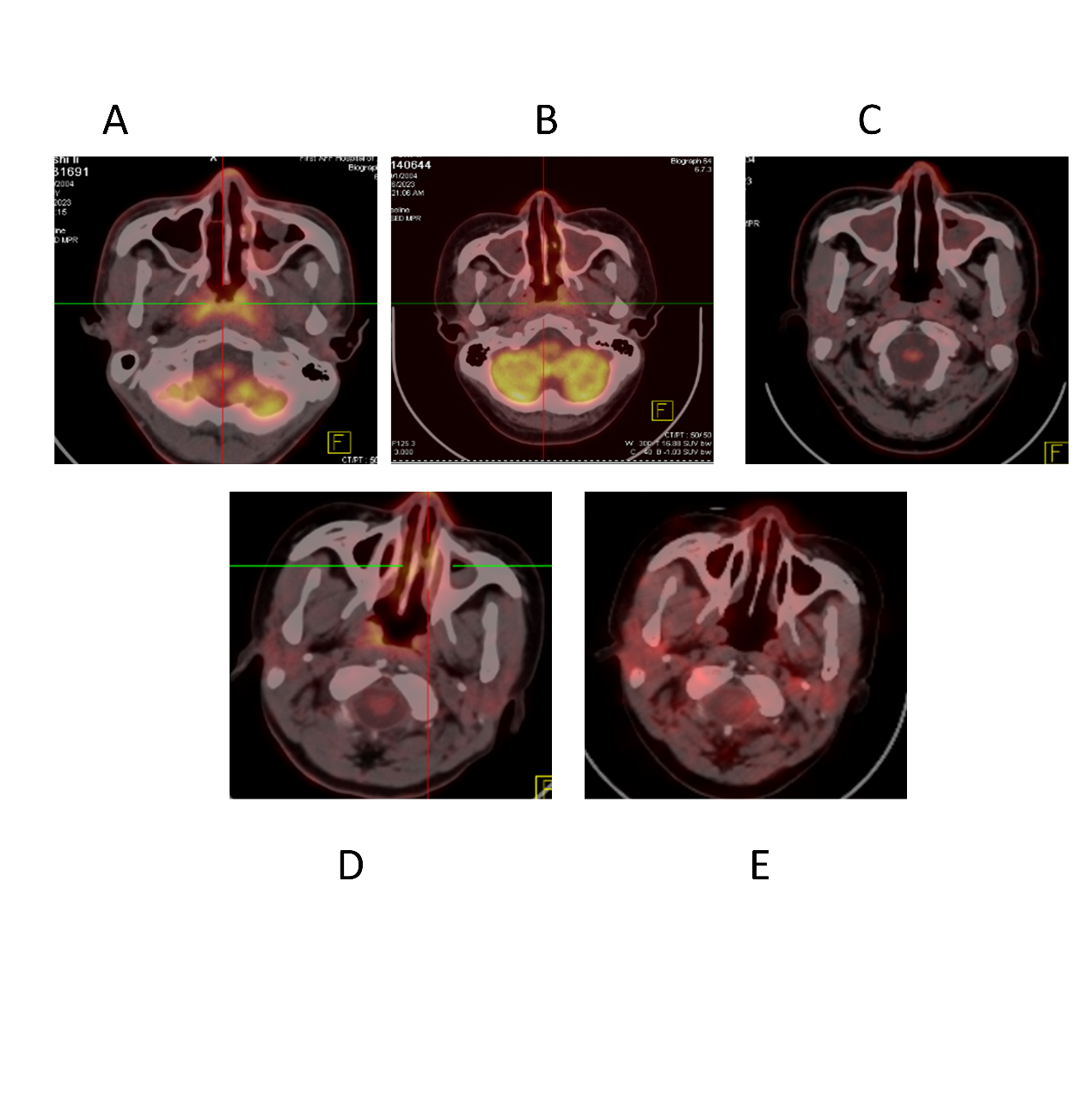


Case 1: (A) Pre-chemotherapy: SUVmax approximately 10.8. (B) Post-chemotherapy: SUVmax approximately 7.6. (C) Three months after CAR-T therapy: SUVmax approximately 2.8.

Case 2: (D) Pre-chemotherapy: Bilateral nasal cavity SUVmax approximately 6.3, nasopharyngeal approximately 8.3. (E) Post-chemotherapy: FDG uptake decreased to normal levels.

Figure 2: Remaining images from the first immunohistochemical staining of Case 1.


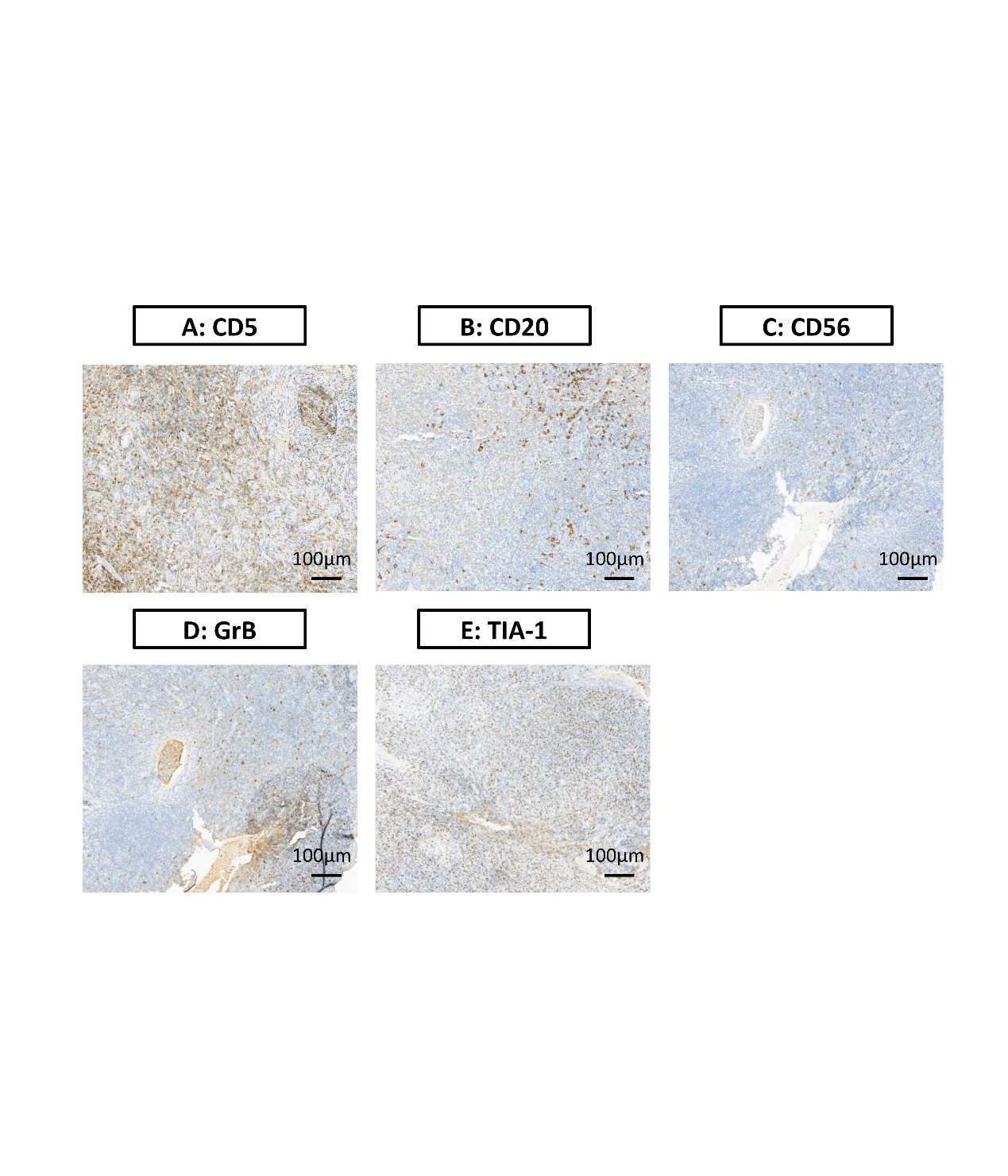
 (A-D) Partially positive for CD5, CD20, CD56, GrB (E) Positive for TIA-1.

Figure 3: H-E and immunohistochemical staining of the second biopsy in Case 1.


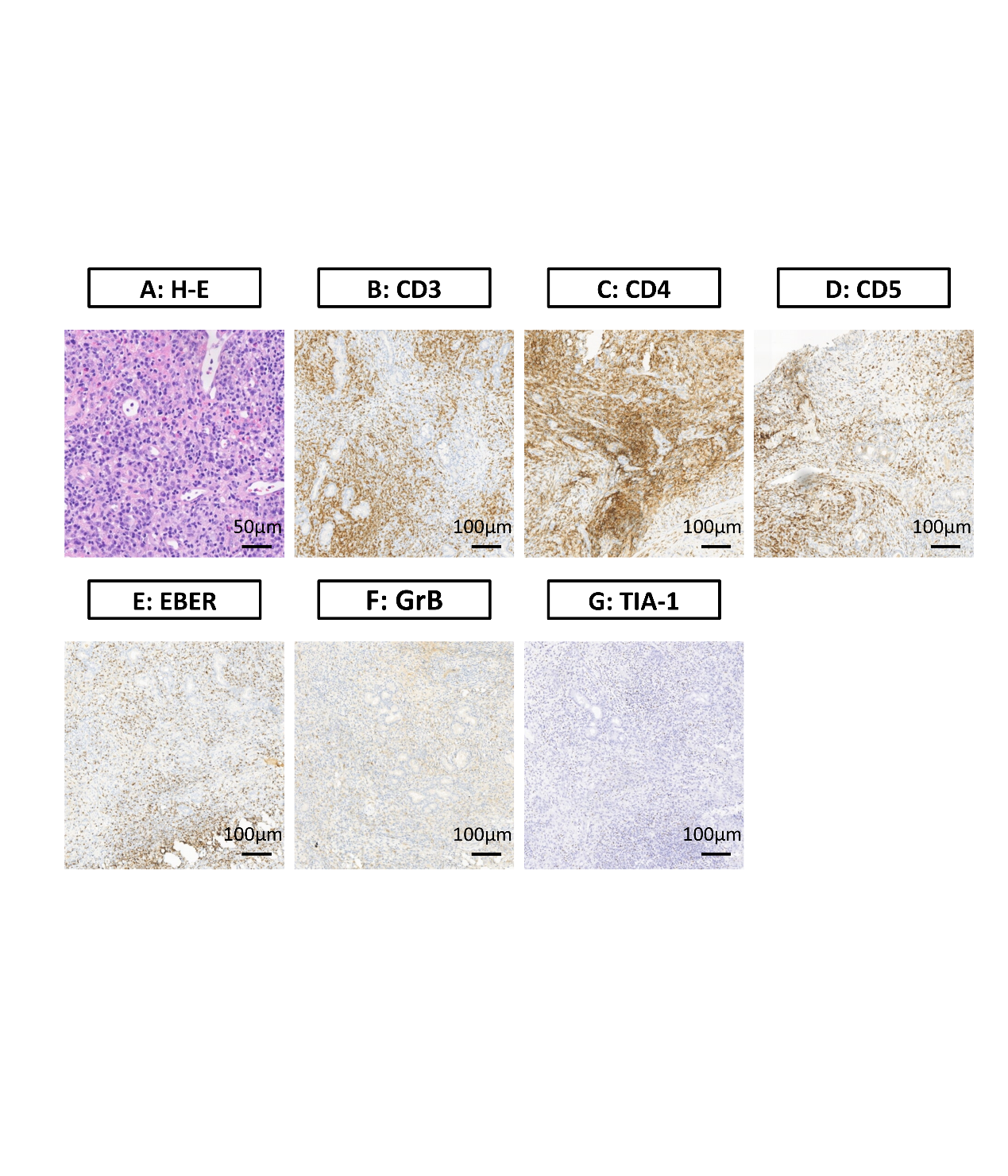


(A) H-E staining shows a large number of lymphocytic infiltrates. (B, C, D, G) Positive for CD3, CD4, CD5, TIA-1. (E) EBER in situ hybridization is positive. (F) Partially positive for GrB.

Figure 4: Remaining images from the immunohistochemical staining of Case 2.


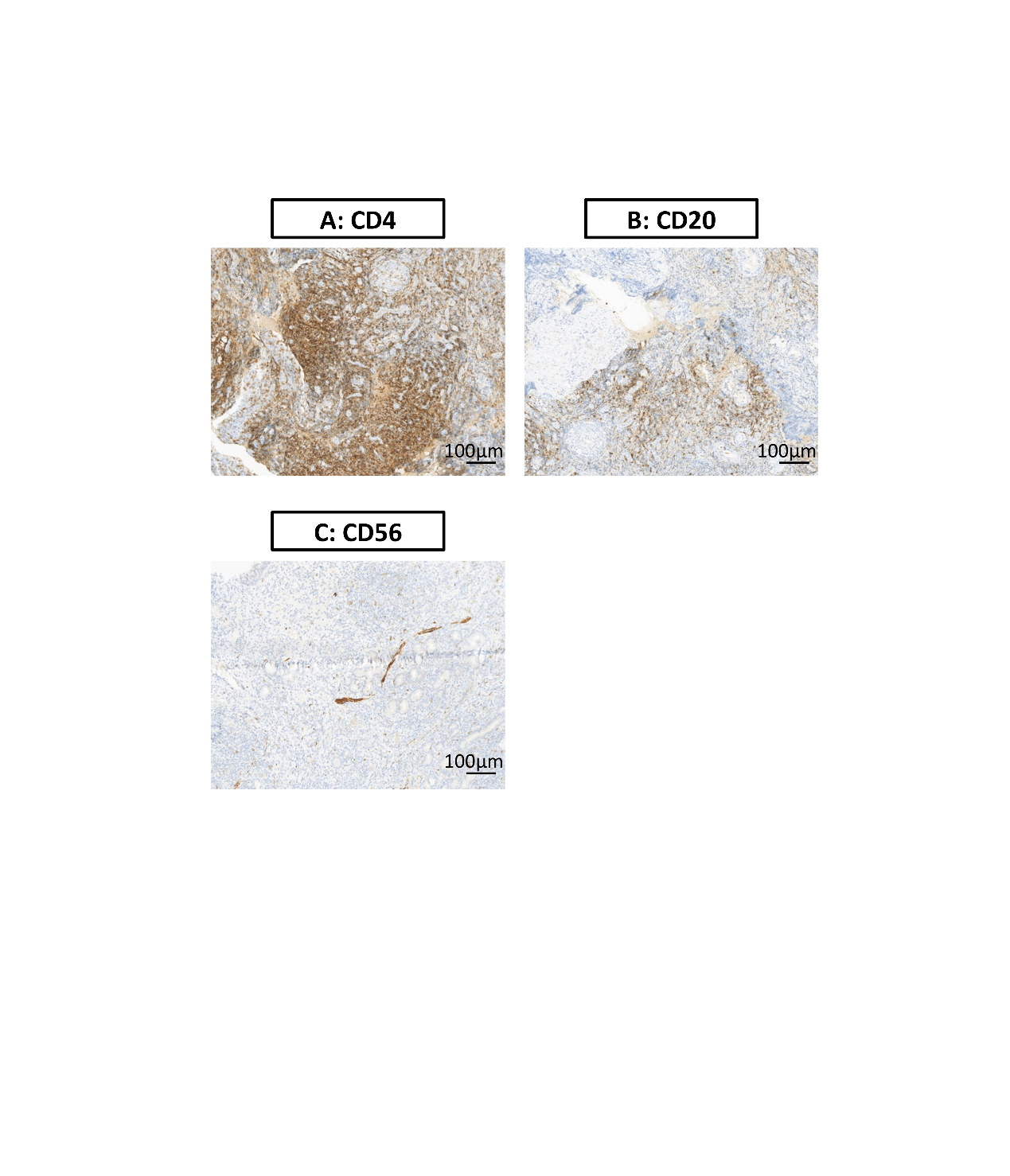
(A) Positive for CD4. (B, C) Partially positive for CD20, CD56.

|  | CAEBV | ENKTL |
| --- | --- | --- |
| Lesion site | The lesion has an extensive range and can infiltrate multiple sites. It mainly involves the liver, spleen, and lymphoid tissues of the nasopharynx. | Mostly occurs in the midline of the face, especially in the nasal cavity. Lymph node infiltration is rare in the early stage, can involve ipsilateral cervical lymph nodes in the progressive stage, and can involve distant sites or the whole body in the advanced stage. |
| Appearance of the lesion | Inflammatory changes with no obvious mucosal ulceration. | Severe mucosal ulceration, with bone destruction in severe cases. |
| Progression of the disease | Systemic symptoms such as fever since the onset of the disease, which then recurred chronically and gradually worsened. | Start with local symptoms, and systemic complications occur in the advanced stage. |
| Histological morphology | Mainly characterized by chronic inflammatory infiltration, without obvious vascular destruction, coagulative necrosis, or obvious malignant features. | Angiocentric growth with extensive vascular destruction and coagulative necrosis, accompanied by significant cellular atypia. |
| Molecular immunology | Most express T-cell markers such as CD3 and TIA-1, and a few are positive for CD56. | Expresses NK cell marker CD56, and partially expresses T cell markers. |
| Relationship with EBV | Persistent infection of T/NK cells leads to dysregulation of the host's immune function. Some patients may progress to lymphoma or leukemia. | Viral proteins such as LMP1 directly drive malignant transformation through pathways like NF-κB/JAK-STAT, including promoting the proliferation of tumor cells and immune evasion. |

Table 1 Differential diagnosis between CAEBV and ENKTL.
